# Effects of *Bacopa monnieri* herbal supplement on aging and neurocognitive functions, including neurophysiological assessments, in relation to constitution (*Prakriti*) in healthy adults: clinical trial protocol

**DOI:** 10.3389/fmed.2026.1702773

**Published:** 2026-02-26

**Authors:** H. L. N. R. Pradeep, P. K. Perera, P. R. Waratenne, N. Samaranayake, W. D. N. Dissanayake

**Affiliations:** 1 Department of Basic Principles, Ayurveda Anatomy and Physiology, Faculty of Indigenous Medicine, University of Colombo, Colombo, Sri Lanka; 2 Department of Ayurveda Pharmacology, Pharmaceutics and Community Medicine, Faculty of Indigenous Medicine, University of Colombo, Colombo, Sri Lanka; 3 Department of Parasitology, Faculty of Medicine, University of Colombo, Colombo, Sri Lanka; 4 Department of Physiology, Faculty of Medicine, University of Colombo, Colombo, Sri Lanka

**Keywords:** Bacopa monnieri, anti-aging, neurocognition, *Prakriti* (Ayurvedic constitution), telomere length, telomerase activity, nerve conduction test (NCT), electroencephalography (EEG)

## Abstract

**Background:**

Aging is an irreversible process shaped by genetic and environmental factors. Beyond physical and mental decline, it involves complex biological mechanisms. Programmed theories propose aging follows a biological clock, while error theories attribute it to environmental damage. A key mechanism is telomere shortening; once critically shortened, cells enter senescence. Telomerase preserves telomeres, but its activity decreases with age. Aging also causes cognitive decline, measurable through tools like electroencephalography (EEG).

**Study design:**

This two-arm, double blind, placebo-controlled superiority randomized controlled trial evaluates the effects of *Bacopa monnieri* freeze-dried herbal decoction (BMFD) on aging and cognition. Elderly participants (MoCA ≥24) will be randomized to receive BMFD or placebo, administered as 120 mL twice daily for 45 days, with assessments at baseline, 45, and 90 days. Conducted at the Clinical Trial Center, Faculty of Indigenous Medicine, University of Colombo, the trial tests BMFD’s ability to stabilize aging processes and enhance neurocognitive functions.

**Outcomes:**

Primary outcomes include telomerase activity and telomere length (qPCR). Secondary outcomes include neurocognitive function [validated MoCA for the Sri Lankan population], neurophysiological functions—nerve conduction test (NCT) for upper and lower limb nerves and brain electrical activity (EEG)—and assessing health-related quality of life (HRQoL) using a validated questionnaire.

**Discussion:**

This trial will provide evidence on BMFD’s potential to preserve telomere length, support cognitive functions, and slow aging processes. Integrating Ayurvedic principles, including constitution (*Prakriti*), it aims to enable early detection of cognitive decline and aging-related changes. Findings are expected to inform preventive and personalized strategies, contributing to evidence-based applications of *Bacopa monnieri* in healthy aging.

**Clinical trial registration:**

https://doi.org/10.1186/ISRCTN64126920, identifier ISRCTN64126920.

## Introduction

### Aging: a multifactorial and irreversible process

Aging is an irreversible, multifactorial process influenced by both intrinsic (genetic) and extrinsic (environmental) factors, contributing to individual variability in longevity (1, 2). It is not merely a decline in bodily functions but a complex mechanism. Theories of aging include programmed theories, which suggest regulation by biological clocks, and error theories, attributing aging to gradual accumulation of DNA damage, free radicals, and macromolecular cross-linking (2). Cellular senescence, resulting from telomere shortening (replicative senescence) or cell stress (cellular senescence), is closely associated with aging (2). Recent studies indicate that aging cannot be captured by a single biomarker and requires integrative assessment of biological, functional, and behavioral parameters. A multicenter frailty-detection study using machine-learning approaches emphasized the importance of combining molecular and functional markers for predictive modeling (3), aligning with the present protocol’s evaluation of telomere biology, neurocognitive function, electrophysiology, and constitution (*Prakriti*).

### The role of telomeres in aging

Telomeres are specialized structures at the ends of chromosomes, composed of clusters of G residues (4). They maintain chromosomal stability by ensuring complete replication of DNA during cell division (5). Telomeres shorten with each division, leading to replicative senescence. Critically short telomeres result in chromosome degradation and progressive genetic loss. Telomerase, a specialized enzyme, helps maintain telomere length and prevent cellular senescence (6–8). Despite its importance, telomerase activity declines with age (9–12) and is often undetectable in many human cells, though it remains measurable in germline cells, cancer cells, and actively dividing peripheral blood mononuclear cells (13–15).

### Aging and *Prakriti*

*Prakriti*, or a person’s tridosha-based constitution, influences susceptibility to chronic diseases (16–18). While well-established in Ayurveda, modern science increasingly recognizes these links. Disease risks can be predicted based on body types: *Kapha*-dominant individuals are prone to weight gain and metabolic syndrome (heart disease, hypertension, diabetes); *Pitta*-dominant individuals to ulcers, bleeding disorders, and skin conditions; *Vata*-dominant individuals to neurological issues, dementia, movement and speech disorders, arrhythmias, and related chronic diseases (19–24). Classical texts suggest *Vata* types have the highest overall disease risk. Long-term cohort studies also show that environmental and climatic factors influence chronic physiological processes and pain sensitivity with age (25), aligning with Ayurveda’s recognition of individual variability in response to environmental stressors, metabolism, and aging.

### Cognitive decline in aging

Aging is often accompanied by cognitive decline. Synchronous neural firing, generating rhythmic brain oscillations, can be measured with scalp EEG (26–28). Parameters such as power, peak frequency, and phase correlate with neurocognitive functions (29–31). Changes in these oscillatory parameters with age provide insights into the evolution of cognitive function over time (32).

### Health implications of aging

Increased life expectancy poses challenges related to age-associated health issues. Common causes of death in older adults include respiratory diseases, heart disease, cancer, and stroke, while chronic conditions such as arthritis, diabetes, osteoporosis, Alzheimer’s disease, depression, Parkinson’s disease, and age-related urinary problems are prevalent (33). Approximately two-thirds of daily global deaths (~150,000) are age-related (34). The growing elderly population (≥65 years) increases strain on healthcare systems, pension funds, and the workforce, with regional disparities further compounding these challenges (35, 36).

### Sensory and motor impairments in aging

Peripheral neuronal weakening in aging affects sensation, muscle strength, balance, and gait. Declining nerve transmission reduces tactile sensitivity and contributes to visual and hearing impairments. Reduced neuromuscular efficiency weakens coordination, shortens reaction times, and increases the risk of falls and mobility issues. Assistive devices can partially compensate, but age-related motor and balance decline remain challenging. Neurological diseases such as Parkinson’s disease can accelerate deterioration. An individual-participant meta-analysis showed that quantitative sensory and neurophysiological markers strongly predict outcomes in chronic degenerative conditions (37), supporting the use of nerve conduction studies and EEG in this trial to capture age-related changes in neuroplasticity and neural efficiency relevant to cognitive aging.

### The role of *Bacopa monnieri* in cognitive health

*Bacopa monnieri*, a traditional Ayurvedic herb, has long been used to enhance memory, learning, and overall cognitive function. It also exhibits sedative and anti-epileptic properties (38). Despite its historical use, scientific evidence on *Bacopa monnieri* decoctions, such as BMFD, for stabilizing aging and cognitive decline is limited. While memory-enhancing effects are recognized, there is no definitive evidence on its impact on telomerase activity, telomere length, or neurocognitive functions in older adults (39). Telomerase activity and telomere length can be measured in blood mononuclear cells, whereas cognitive function requires neuropsychological assessment. Recent studies indicate that gut microbiota composition and tryptophan-derived metabolites influence neuroinflammation, pain regulation, and cognition via the gut–brain axis (40). *Bacopa monnieri*’s antioxidative, anti-inflammatory, and neuroprotective properties may underlie its potential effects on telomere stability and cognitive resilience in aging adults.

### Research gaps and study purpose

This study aims to evaluate the potential of BMFD decoction in stabilizing aging and influencing neurocognitive functions in the elderly. Evidence on its effects on telomerase activity, telomere length, and age-related cognitive changes—particularly in relation to Deha Prakriti (body constitution)—is limited. By examining these effects, this study seeks to explore how BMFD may support healthy aging and neurocognitive functions, contributing to personalized interventions, including dietary, lifestyle, and herbal strategies, to mitigate cognitive decline and enhance overall well-being.

## Methods

### Study design

This is a two-arm, open-label, non-inferiority randomized controlled clinical trial that will be conducted at the Clinical Trial Center, Faculty of Indigenous Medicine, University of Colombo, Sri Lanka. The test product will be BMFD, a freeze-dried formulation of *Bacopa monnieri*. The trial will evaluate the effects of the *Bacopa monnieri* herbal supplement on aging, neurocognitive functions with relation to Constitution (*Prakriti*), and constitution (*Prakriti*) in healthy adults, compared to a placebo. Participants with (the MoCA score of 24 or above) will be randomly assigned to one of two arms following a 1-week run-in period, with the BMFD herbal supplement administered orally for 45 days. This study protocol has been developed in accordance with the Standard Protocol Items: Recommendations for Interventional Trials (SPIRIT) guidelines. Ethics approval has been obtained from the Ethics Review Committee, Faculty of Indigenous Medicine (ERCFIM), University of Colombo, Sri Lanka (ERC-23/202). The trial is registered in the ISRCTN registry under trial number ISRCTN64126920 https://doi.org/10.1186/ISRCTN64126920.

### Study setting

The study will be conducted at the Clinical Trial Center, Faculty of Indigenous Medicine, University of Colombo, Sri Lanka. Participants will be recruited from healthy individuals, with the MoCA score of 24 or above. Recruitment will take place through a newspaper advertisement inviting individuals to participate in the trial at the Clinical Trial Center, Faculty of Indigenous Medicine, University of Colombo.

### Participants

Participation in this research project is voluntary. Participants will be recruited through a screening process to assess eligibility based on inclusion and exclusion criteria. Eligible individuals will then be randomly assigned to either the BMFD decoction group or the placebo group.

### Inclusion and exclusion criteria

The inclusion criteria include: (1) healthy individuals aged between 60 and 64 years at the time of enrollment, of either sex; (2) nonsmokers and non-alcoholic; (3) the MoCA (the Montreal Cognitive Assessment) score of 24 or above. Participants will not be permitted to take any other medications during the trial period. If they need to take any additional medication, they must notify the investigators and withdraw from the trial. The exclusion criteria include: (1) individuals with Chronic diseases, e.g., Kidney Disease, Cardiovascular Disease, Liver disorders, psychiatric illnesses, Diabetes Mellitus, cancers, Hypertension, hyperlipidemia; (2) participants with dyspeptic symptoms; (3) those with reduced capacity to complete the tasks involved in the study.

### Assessment of telomere length and telomerase activity

Telomere length will be assessed using a quantitative PCR (qPCR)–based assay, and telomerase activity will be evaluated using the telomeric repeat amplification protocol (TRAP) assay, in accordance with SPIRIT recommendations. Peripheral blood mononuclear cells (PBMCs) will be isolated from collected blood samples and processed under standardized laboratory conditions. DNA and protein will be extracted using validated protocols, and commercially available assay kits from the respective manufacturers will be employed. For the qPCR assay, specific primer sequences and appropriate reference genes will be used, with plate layout, internal controls, calibration procedures, and standard curve generation clearly defined. Reaction conditions and cycling parameters will be standardized across all runs. Quality assurance procedures will include duplicate or triplicate measurements, evaluation of intra- and inter-assay variability, and predefined criteria for repeating assays. Telomerase activity will be quantified using a TRAP-ELISA–based approach, incorporating positive and negative controls, with results analyzed using a defined quantification and normalization strategy. These procedures ensure methodological rigor, transparency, and reproducibility of biomarker assessment within the trial.

### Sample size

The primary outcomes of this clinical trial are telomere length, measured in kilobases using quantitative PCR, and telomerase activity, quantified using an ELISA-based TRAP assay. Both are continuous biological markers that reflect cellular aging processes. Sample size estimation was conducted using the formula for comparing two independent means, appropriate for continuous outcomes. Based on pilot findings reported by Ornish et al. (41), a mean difference of 0.5 kb in telomere length between groups was considered clinically relevant, with a standard deviation (SD) of 0.7 kb. A significance level of *α* = 0.05 and power of 80% (*β* = 0.20) were applied in the calculation. Using these parameters, the required sample size per group was obtained, and after accounting for a 10% dropout rate, the final required sample size was determined to be 37 participants per group, resulting in a total of 74 participants for the study. Although Ornish et al. (41) studied men with prostate cancer—a different population from our healthy older adults—we used this data as it provided the most relevant preliminary estimate of variability and effect size available. We acknowledge that this may limit the precision of our sample size calculation and will interpret our findings accordingly. With a significance level of *α* = 0.05 and power of 80% (*β* = 0.20), the required sample size per group was calculated, and after accounting for a 10% dropout rate, the final sample size was determined to be 37 participants per group, for a total of 74 participants. To further strengthen the validity of these assumptions, a dose-finding and feasibility phase will be conducted prior to the full RCT. Data generated from this phase will be used to validate and refine the expected effect sizes, particularly for telomerase activity and cognitive outcomes, thereby enhancing the robustness of the final trial estimates.

### Recruitment

Individuals interested in participating in this clinical study will receive a detailed Patient Information Leaflet (PIL), which will be accompanied by a verbal explanation of the study procedures. If participants agree with the information provided in the PIL, they will then complete a screening questionnaire. Written informed consent will be obtained from each participant by the investigators prior to the initial interview. During the initial interview, participants will undergo a comprehensive history taking, physical examination, and various hematological and biochemical investigations (including FBS, FBC, ESR, ALT/AST, serum creatinine, and UFR). Recruitment based on MoCA analysis score [the MoCA score of 24 or above (42–47)]. Participants who meet the inclusion and exclusion criteria will be recruited for the study. All baseline assessment forms [including the MoCA analysis form, *Prakriti* analysis (constitution), Quality of Life questionnaire] will be completed by the investigators. Participants will not be permitted to take any other medications during the trial period. If they need to take any additional medications, they must inform the investigators and discontinue participation in the trial.

### Baseline assessment

The Montreal Cognitive Assessment (MoCA) test, the Nerve Conduction Test (NCT), electroencephalography (EEG), constitutional analysis, and health-related quality of life (HRQoL) will be assessed at baseline. Additionally, hematological and biochemical investigations, including fasting blood sugar (FBS), full blood count (FBC), erythrocyte sedimentation rate (ESR), alanine aminotransferase/aspartate aminotransferase (ALT/AST), serum creatinine, and urine full report (UFR), will be conducted.

### Randomization

**Figure 1:**
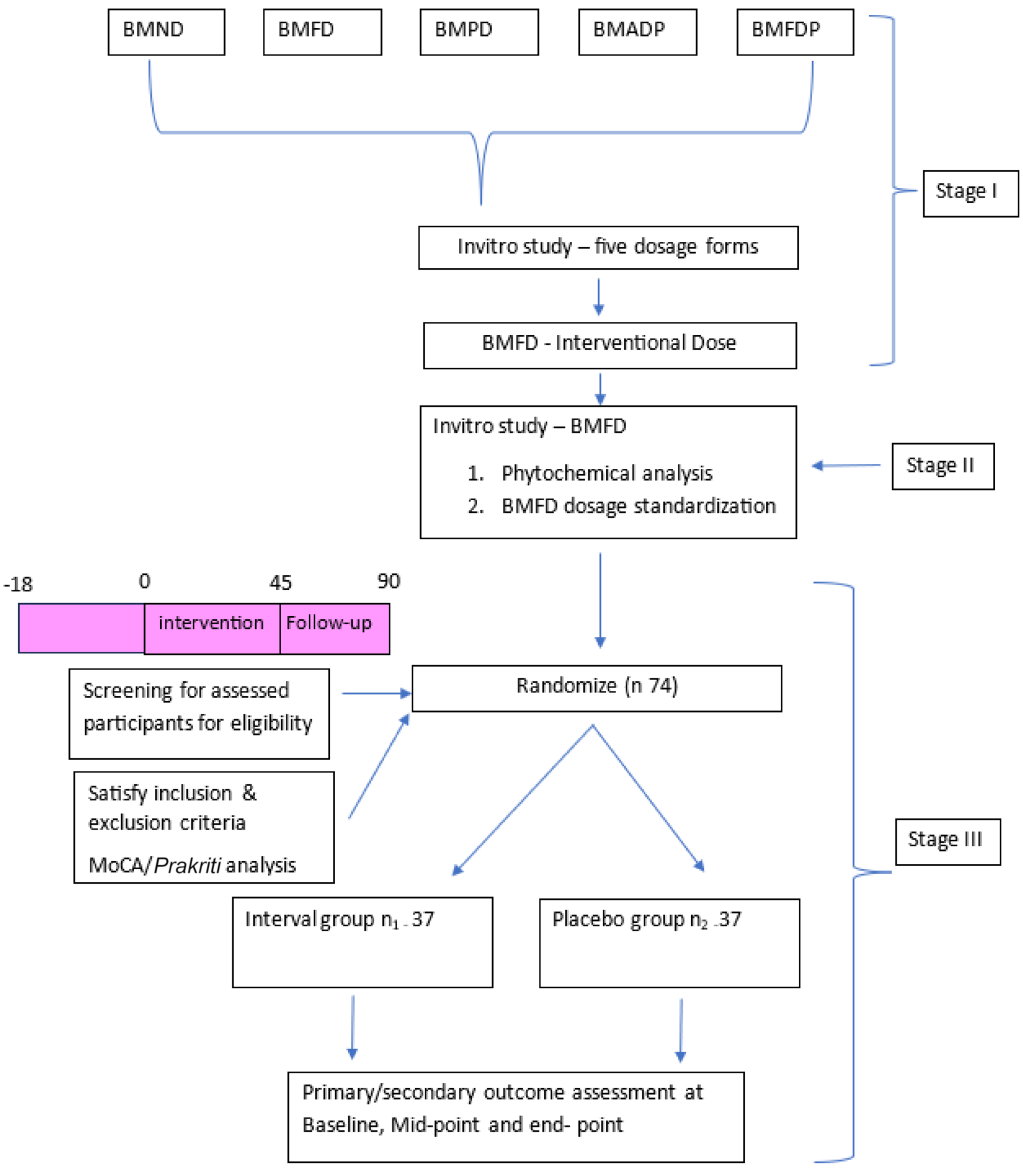
Study design. BMND; *Bacopa monnieri* (L.) Wettst normal decoction, BMFD; *Bacopa monnieri* (L.) Wettst freeze dried decoction, BMPD; *Bacopa monnieri* (L.) Wettst phanta decoction (hot infusion). BMD; *Bacopa monnieri* (L.) Wettst freeze dry powder, BMFDP; *Bacopa monnieri* (L.) Wettst freeze dry powder.

The randomization sequence will be generated using an online randomization tool.^1^ Block randomization will be applied with blocks of 12 to create a randomization schedule for 74 participants. Patients will be assigned to treatment groups based on the generated sequence. A one-week supply of the assigned investigational product will be distributed to the patients according to their random allocation. The allocation ratio will be 1:1 for each group. Each randomization number’s corresponding allocation will be placed into individually sealed, opaque envelopes. These envelopes, along with the allocation sequence, will be securely stored by an investigator who is not involved in participant recruitment. Participants who meet the inclusion and exclusion criteria will be enrolled in the study and assigned a randomization number sequentially, based on the date and time of their recruitment. The treatment indicated in the sealed envelope for each number will then be provided to the corresponding participant. A flowchart depicting the study design is presented in Figure 1.

## Intervention

### Standardization of dosage

The dosage of *Bacopa monnieri* for the main trial will be standardized based on a preliminary pilot dosing study. This pilot study will involve administering different doses to a small group of participants to evaluate safety, tolerability, and preliminary efficacy. Data collected from this phase will inform the selection of the optimal dose that balances efficacy with safety. Additionally, the selected dose will be supported by phytochemical analysis, ensuring consistency of active constituents such as bacoside A in the prepared decoction. This approach ensures that the dosage used in the main trial is evidence-based, safe, and reproducible.

### Intervention *Bacopa moneri* herbal supplement (BMFD)—Arm 1

BMFD was prepared following the traditional method for decoction preparation (48). Sixty grams of freeze-dried *Bacopa monnieri* (L.) Wettst was boiled with 1920 mL of water over a mild flame until the volume reduced to 240 mL. The decoction was then filtered through a single-folded cotton cloth and collected in a separate vessel. A 60-gram pack of freeze-dried *Bacopa monnieri* (BMFD) will be used to prepare the decoction required for 1 day. A one-week supply, consisting of seven packs, will be provided to each participant. Patients will be instructed to place the supplied herbal pack into a pot, add 1920 mL of water, and simmer it over low heat until the volume is reduced to 240 mL. The preparation process, carried out under standardized conditions, will be demonstrated to participants assigned to the BMFD arm at the clinical trial center of the Faculty of Indigenous Medicine (FIM) through a video. Participants will be instructed to consume a daily dose of 120 mL, taken twice a day before meals.

### Placebo—Arm 2

To prepare the placebo while minimizing antioxidant content, freeze-dried green tea leaves will undergo a repeated steeping process. First, the leaves will be placed in 1920 mL of boiling water and simmered for 30–60 min, after which the liquid will be discarded. This process will be repeated two to three times with fresh water to further reduce the antioxidant content. Afterward, the treated leaves will be freeze-dried to restore their original form. A 60-gram pack of these processed freeze-dried green tea leaves will be used to prepare the placebo decoction for 1 day. The preparation process, carried out under standardized conditions, will be demonstrated to participants in the placebo group at the clinical trial center of FIM through a video. Participants will be instructed to consume a daily dose of 120 mL, taken twice a day before meals. The placebo will be prepared from repeatedly boiled green tea leaves to minimize the presence of caffeine and other bioactive constituents. Although trace amounts may remain, their concentrations will be expected to be negligible and unlikely to produce meaningful cognitive or physiological effects. This method will provide a practical and ethically acceptable placebo that closely matches the appearance and taste of the active preparation while maintaining minimal biological activity.

### Placebo preparation and phytochemical verification

To ensure the validity of the placebo and confirm its phytochemical inertness, the final placebo batch will undergo analytical verification prior to administration. Total phenolic content and antioxidant activity will be assessed using standardized assays such as DPPH and FRAP. Only batches demonstrating negligible or non-detectable antioxidant activity will be approved for use, thereby minimizing any potential biological effects that could influence study outcomes.

The placebo will be prepared using freeze-dried green tea leaves subjected to repeated steeping and exhaustive extraction, a process designed to remove water-soluble polyphenols, flavonoids, and other bioactive constituents while retaining the inert plant matrix. This procedure ensures that the placebo remains biologically inactive while maintaining physical consistency.

To preserve blinding integrity, the placebo will be matched to the BMFD intervention in terms of color, texture, aroma, and mode of preparation. Both BMFD and placebo will be supplied as identically packaged sachets, prepared using the same reconstitution protocol, and presented to participants in a standardized manner to minimize expectancy bias.

### Placebo preparation and justification

The placebo, prepared from repeatedly boiled freeze-dried green tea leaves, has been chosen because it provides a similar color, appearance, and aroma profile to BMFD. To address concerns about residual bioactive components, the final placebo batch will undergo analytical confirmation to ensure that any remaining antioxidant activity or caffeine content is negligible. This process supports the validity of the placebo as an inert control while maintaining blinding integrity.

### Storage, packaging, and dispensing of investigational herbal supplement and placebo

Both products (BMFD and placebo) will be packaged for a 7-day supply and labeled with relevant details, including the batch number, dosage, time of administration, and mode of administration. These packages will be securely stored at the clinical trial center of the Faculty of Indigenous Medicine (FIM), University of Colombo, Sri Lanka, and will be dispensed to participants based on the predetermined randomization sequence. A 7-day supply of the investigational products will be provided to study participants during their weekly visits, along with appropriate instructions (see Table 1).

**Table 1:** Investigational products.

Herbal supplement	Dose	Mode of administration	Route	Method of preparation
BMFD	120 mL	Morning and evening before meals	Oral	60 g (BMFD) of freeze-dried leaves of plant materials boiled with 1920 mL of water and reduced to 240 ml
Placebo	120 mL	Morning and evening before meals	Oral	Placebo

### Storage, packaging, and dispensing of investigational supplement

#### Outcome measurements

##### Primary outcomes

The following primary outcome measures is assessed at baseline, 45 and 90 days:

Telomerase activity and telomerase length of blood mononuclear cells of participants measured using qualitative polymerase chain reaction (QPCR).

The following secondary outcomes measures are assessed at baseline, 45 and 90 days:

##### Secondary outcomes

1. Neurocognitive function [validated MoCA for the Sri Lankan population—the Sinhala version of the Montreal Cognitive Assessment (MoCA-S) was validated and published in 2011 as a screening instrument for dementia in Sri Lanka]. 2. Neurophysiological functions—peripheral nerve conduction (upper and lower limb nerves). 3. Brain electrical activity (EEG). EEG data will be collected at baseline, at mid-point and follow-up visits to assess neurophysiological changes associated with aging. Resting-state EEG recordings will be obtained with participants seated comfortably, using a 64-channel cap following the 10–20 system. Participants will undergo 5-min recordings with eyes open and eyes closed. The raw EEG data will be preprocessed by applying band-pass filtering (0.5–45 Hz), notch filtering at 50/60 Hz, and artifact removal through automated detection, manual review, and independent component analysis to eliminate eye blinks, muscle activity, and other noise. Key parameters such as spectral power in delta, theta, alpha, beta, and gamma bands, peak frequency, and measures of signal complexity will be extracted. Connectivity analyses, including coherence and phase lag indices, will also be performed. These neurophysiological metrics will be analyzed to evaluate age-related brain changes and their association with clinical outcomes. 4. Assess health-related quality of life (HRQoL) using a validated questionnaire and Health-related quality of life (HRQoL) measured using an HRQoL questionnaire (52).

### Safety assessment

Each participant will undergo hematological and biochemical assessments, including fasting blood sugar (FBS), full blood count (FBC), erythrocyte sedimentation rate (ESR), aspartate aminotransferase (AST), alanine aminotransferase (ALT), and serum creatinine/glomerular filtration rate (GFR), along with a comprehensive urine analysis, both before and after the intervention. These evaluations primarily serve as a safety assessment measure. Adverse events experienced by participants will be systematically recorded on a weekly basis by investigators during scheduled visits to the clinical trial center. Additionally, participants will be instructed to document any adverse reactions in their diaries and promptly report them using the designated contact numbers. In the event of unexpected symptoms or concerns, they will be advised to visit the trial center at the Faculty of Indigenous Medicine for further evaluation. Any serious adverse events will undergo thorough assessment and will be reported to the Ethics Review Committee (ERC) of the Faculty of Indigenous Medicine within five working days. Although no severe adverse reactions are anticipated with BMFD Herbal supplement, in cases where an adverse reaction necessitates hospitalization, appropriate medical care and expert management will be ensured. If at any point the safety of trial participants can no longer be guaranteed or new scientific evidence emerges indicating potential risks, the clinical trial will be prematurely terminated.

#### Safety monitoring and stopping criteria

Participants will be monitored for adverse events throughout the study period. Predefined laboratory thresholds for discontinuation include elevations of liver enzymes (ALT or AST > 3× upper limit of normal), serum creatinine >1.5× baseline, or any laboratory abnormality graded ≥3 according to CTCAE criteria. Occurrence of any serious adverse event deemed related to the intervention will result in immediate discontinuation of the study product for that participant. The trial will be temporarily suspended if two or more participants experience unexpected serious adverse events related to the intervention, pending safety review.

### Blinding procedure

To ensure double blinding, an independent staff member (unblinded dispenser), who is not involved in participant recruitment, outcome assessment, or data analysis, will be responsible for labeling, coding, and dispensing either BMFD or placebo sachets according to the randomization schedule. The PI, investigators, outcome assessors, and participants will remain blinded to group allocation until completion of the trial. Emergency unblinding will be permitted only if medically necessary.

### Outcome assessor blinding

All primary and secondary outcomes will be measured in both the BMFD and placebo groups using standardized procedures.

Primary outcomes (telomerase activity and telomere length) will be measured using laboratory assays with coded samples, ensuring objective assessment and minimizing bias.

Secondary outcomes [cognitive function, neurophysiological function, and health-related quality of life (HRQoL)] will be administered identically to both groups. Where feasible, scoring of subjective outcomes will be performed by assessors blinded to group allocation to reduce potential measurement bias (see Table 2).

**Table 2:** Study procedures.

Study procedures/assessments	Screening (Week 1)	Baseline (Week 0)	Week 6	Week 12 (Endline)
Enrollment				
Eligibility screening	✓			
Informed consent	✓			
Recruitment	✓			
Randomization		✓		
Interventions				
ARM 1: BMFD administration		✓	✓	✓
ARM 2: Placebo administration		✓	✓	✓
Assessments/investigations				
MoCA (cognitive assessment)		✓	✓	✓
Constitutional (*Prakriti*) analysis		✓		
Nerve conduction test (NCT)		✓		✓
Electroencephalography (EEG)		✓		✓
Telomere length		✓		✓
Telomerase activity		✓		✓
Health-related quality of life (HRQoL)		✓	✓	✓
Safety monitoring				
Adverse event assessment		✓	✓	✓

✓ indicates the procedure performed at the specified timepoint.

Baseline = prior to initiation of intervention.

Week 12 represents end-of-treatment follow-up.

HRQoL—health-related quality of life.

### Data handling, record keeping, and dissemination

Each participant will have an individual file to securely store hard copies of case record forms, including informed consent, results from hematological and biochemical investigations, physical examination findings, completed questionnaires, NCT and EEG Data, Moca test data and QPCR analysis data. Data entry will be performed by a limited number of dedicated staff members and saved on a password-protected, dedicated computer. These data will remain with the researchers and will not be shared with any third party under any circumstances. Participant information will be securely stored during each clinic visit throughout the study. Upon study completion, all records will be securely retained for a period of 10 years. Participant data will be stored at the Department of Basic Principles, Ayurveda Anatomy, and Physiology, Faculty of Indigenous Medicine, University of Colombo. These data will be used exclusively for statistical analysis and scientific reporting. Identifying information, such as contact details, will be stored separately from research data. Each participant and their corresponding data will be assigned a unique study identification number. After the study concludes, all databases will be de-identified and archived. A Data Safety Monitoring Board, composed of three independent experts, has been appointed in accordance with the guidelines set by the Ethics Review Committee of the Faculty of Indigenous Medicine to ensure safety monitoring. As this is a single-center trial involving only 74 participants, no audit is planned for this study. The study results will be disseminated through scientific conferences and peer-reviewed journals. Additionally, individual participants will receive their study results and group allocation information upon the trial’s completion.

### Ethical considerations

The research project protocol has received approval from the Research and Higher Degrees Committee of the Faculty of Medicine, University of Colombo, as well as the Ethics Review Committee of the Faculty of Indigenous Medicine (FIM), University of Colombo. The trial has been registered with the ISRCTN registry under the trial number [ISRCTN64126920—(https://doi.org/10.1186/ISRCTN64126920)]. The study will be conducted in strict adherence to Good Clinical Practice (GCP) guidelines. Any protocol modifications will be submitted to the Ethics Review Committees and updated in the trial registry for approval. Participants will be provided with an information sheet detailing the research in Sinhala, Tamil, and English, and written informed consent will be obtained prior to participation. The information will include the study’s purpose, duration, and potential consequences. Participants will retain the right to withdraw their consent and discontinue participation at any time without any penalty, effect on their medical care, or loss of benefits. The questionnaire will be administered by an interviewer and will remain anonymous. Only essential socio-demographic data, such as occupation and the nature of the participant’s health condition, will be collected. No additional personal data will be gathered by the researchers.

### Data management and ethical oversight

The DSMB will provide independent oversight throughout the trial. Its responsibilities and intervention criteria have been further elaborated in the revised manuscript. Specifically, the DSMB may recommend pausing or terminating the study under the following circumstances:

- Safety-related events, including any serious adverse event (SAE) that is judged to be related to the intervention, unexpected patterns of adverse events, or a significant imbalance in safety outcomes between study arms.
- Recruitment or retention concerns, such as failure to meet predefined recruitment milestones or excessive dropout that threatens scientific validity.
- Protocol deviations that compromise participant safety or data integrity.
- Interim data concerns, should any emerging evidence indicate that continuation poses risk or that the trial objectives cannot be achieved.
- These conditions have now been clearly stated in the ethics section. The long-term data storage plan remains unchanged and aligns with institutional and national guidelines.

### Method of data analysis

For both primary and secondary outcome measures, mean values at baseline and study completion, as well as mean differences, will be compared across the two study arms using ANOVA (Analysis of Variance) or the non-parametric Kruskal–Walli’s test, depending on the data’s normality. Within each treatment arm, changes in primary and secondary outcomes before and after the intervention will be analyzed using either paired-sample *t*-tests or the non-parametric Wilcoxon signed-rank test, based on the distribution of the data. For all normally distributed outcomes, 95% confidence intervals will be calculated. Categorical variables will be analyzed between groups using the Chi-square test. Potential confounding factors will be adjusted using ANCOVA, with adjusted mean values and corresponding confidence intervals subsequently calculated and reported. Regarding missing data, instead of the last observation carried forward, multiple imputation techniques will be employed to handle missing outcome data, ensuring a more robust and unbiased estimation. Statistical analysis will be conducted using the SPSS software (version 20.0), and the level of significance will be set at *α* = 0.05. In cases of missing outcome data, the most recent available values of the outcome measures will be used as replacements. Intention-to-treat analysis will be applied for all efficacy and safety outcomes. Additionally, per-protocol analysis will be conducted for efficacy outcomes, including only participants who complete the follow-up.

### Statistical analysis

The statistical analysis plan has been developed to align with the study objectives and ensure rigorous evaluation of outcomes. Descriptive statistics will be used to summarize baseline characteristics across study arms. Continuous primary and secondary outcomes will be assessed for normality prior to analysis. Between-group comparisons of mean changes from baseline to study completion will be conducted using analysis of variance (ANOVA) for normally distributed data or the Kruskal–Wallis test for non-normal data. Within-group pre–post changes will be analyzed using paired-sample *t*-tests or Wilcoxon signed-rank tests, as appropriate.

To account for baseline imbalances and potential confounding factors such as age, sex, and baseline outcome values, analysis of covariance (ANCOVA) will be applied, with adjusted mean differences reported alongside 95% confidence intervals. Categorical variables will be compared between groups using the Chi-square test or Fisher’s exact test, as appropriate.

Missing outcome data will be handled using multiple imputation methods to minimize bias and improve the robustness of estimates. The primary analysis will follow the intention-to-treat principle for all efficacy and safety outcomes, with a secondary per-protocol analysis conducted for efficacy outcomes among participants who complete the follow-up assessments. All statistical analyses will be performed using SPSS software (version 20.0), with a two-sided significance level set at *α* = 0.05.

## Discussion

As per modern science, aging is the time-dependent, physiological decline of biological processes that are often associated with age-related diseases. Those pathologies often associated with aging, such as cellular inflammation and atherosclerosis, involve hyperactivity or uncontrolled cellular growth. There are about nine hallmarks of aging: genomic instability, telomere attrition, epigenetic alteration, loss of proteostasis, deregulated nutrient sensing, mitochondrial dysfunction, cellular senescence, stem cell exhaustion, and altered intracellular communication (49). The hallmark of anti-aging action is calorie restriction (CR), which involves the extension of the life span and the suppression of age-related diseases. Calorie restriction mechanisms play a major role in modulating chronic inflammation at the molecular level, the impact of epigenetic chromatin and histone modification, and the ultimate control of gene expression (50). Changes in telomere length and telomerase activity could provide valuable insights into the potential influence of *Bacopa monnieri* on aging-related processes. Since telomere shortening is a hallmark of cellular aging and genomic instability, any modulation in its length may indicate an impact on biological aging. Similarly, alterations in telomerase activity could reflect the extent to which *Bacopa monnieri* influences cellular longevity and regenerative capacity. Investigating these changes will help determine whether *Bacopa monnieri* plays a role in slowing down aging-related cellular decline or enhancing genome stability.

Gene sequences called telomeres are found at the ends of chromosomes and are in charge of preserving the integrity of the genome. Telomere length is at its maximum at birth and decreases progressively with advancing age; thus, it is considered a biomarker of chronological aging. Telomerase is the enzyme responsible for maintaining the length of telomeres by adding guanine-rich repetitive sequences. Telomerase activity is exhibited in gametes, stem cells, and tumor cells. Our research team has designed a two-arm, open-label, non-inferiority randomized controlled trial to evaluate the effects of a freeze-dried *Bacopa monnieri* formulation on aging, neurocognitive functions, and constitution (*Prakriti*) in healthy adults, compared to a placebo and his clinical trial will provide evidence-based scientific data on the effectiveness of *Bacopa* and its Ayurvedic dosage form, BMFD, in the aging process and cognitive enhancement concerning individual constitution (*Prakriti*). This trial is expected to contribute to the early diagnosis of neurocognitive impairment and aging-related neurophysiological changes in relation to individual constitution (*Prakriti*). Editorial reflections in aging science emphasize that frailty and cognitive decline represent complex, multidimensional challenges that require integrative biological and socio-cultural strategies (51). By combining traditional Ayurvedic constitutional assessment with modern biomarkers of cellular aging and neurophysiology, this trial contributes to a broader interdisciplinary effort to develop culturally grounded, personalized geriatric interventions.

## Strengths and limitations

This randomized controlled clinical trial evaluates the effects of a freeze-dried formulation of the *Bacopa monnieri* herbal supplement on aging and cognitive function in relation to constitution (*Prakriti*) in healthy adults and multiple outcome measures, including neurocognitive function, telomerase activity, telomere length, nerve conduction tests (NCT), and electroencephalography (EEG), provide a comprehensive assessment of the intervention’s effects. Participants with the MoCA score of 24 or above will be recruited. The findings of this study will offer valuable evidence supporting the use of this herbal preparation in managing age-related neurocognitive decline and neurodegenerative conditions. The study is conducted within a specific population, which may limit the generalizability of the findings.

## Footnotes

1
www.randomisation.com

## References

[1] Kowald A Kirkwood TBL. A network theory of ageing: the interactions of defective mitochondria, aberrant proteins, free radicals and scavengers in the ageing process. Mutat Res. (1996) 316:209–36. doi: 10.1016/s0921-8734(96)90005-3, 8649456

[2] Weinert BT Timiras PS. Invited review: theories of aging. J Appl Physiol. (2003) 95:1706–16. doi: 10.1152/japplphysiol.00288.2003, 12970376

[3] Fernández-Carnero S Martínez-Pozas O Pecos-Martín D Pardo-Gómez A Cuenca-Zaldívar JN Sánchez-Romero EA. Update on the detection of frailty in older adults: a multicenter cohort machine learning-based study protocol. Aging (Albany NY). (2025) 17:1328–39. doi: 10.18632/aging.206254, 40413725 PMC12151518

[4] Blackburn EH. Telomeres: structure and synthesis. J Biol Chem. (1990) 265:5919–21. doi: 10.1016/s0021-9258(19)39264-6, 2180936

[5] Blackburn EH. Telomere states and cell fates. Nature. (2000) 408:53–6. doi: 10.1038/35040500, 11081503

[6] de Lange T. Activation of telomerase in a human tumor. Proc Natl Acad Sci USA. (1994) 91:2882–5. doi: 10.1073/pnas.91.8.2882, 8159672 PMC43476

[7] Wyatt HDM West SC Beattie TL. InTERTpreting telomerase structure and function. Nucleic Acids Res. (2010) 38:5609–22. doi: 10.1093/nar/gkq370, 20460453 PMC2943602

[8] Bodnar AG Ouellette M Frolkis M Holt SE Chiu CP Morin GB . Extension of life-span by introduction of telomerase into normal human cells. Science. (1998) 279:349–52. doi: 10.1126/science.279.5349.349, 9454332

[9] Iwama H Ohyashiki K Ohyashiki JH Hayashi S Yahata N Ando K . Telomeric length and telomerase activity vary with age in peripheral blood cells obtained from normal individuals. Hum Genet. (1998) 102:397–402. doi: 10.1007/s004390050711, 9600234

[10] Mosquera A Fernandez JL Campos A Goyanes VJ Ramiro-Diaz J Gosalvez J. Simultaneous decrease of telomere length and telomerase activity with ageing of human amniotic fluid cells. J Med Genet. (1999) 36:494–6. 10874642 PMC1734391

[11] Son NH Murray S Yanovski J Hodes RJ Weng N. Lineage-specific telomere shortening and unaltered capacity for telomerase expression in human T and B lymphocytes with age. J Immunol. (2000) 165:1191–6. doi: 10.4049/jimmunol.165.3.1191, 10903716

[12] Cherif H Tarry JL Ozanne SE Hales CN. Ageing and telomeres: a study into organ- and gender-specific telomere shortening. Nucleic Acids Res. (2003) 31:1576–83. doi: 10.1093/nar/gkg208, 12595567 PMC149817

[13] Wright WE Piatyszek MA Rainey WE Byrd W Shay JW. Telomerase activity in human germline and embryonic tissues and cells. Dev Genet. (1996) 18:173–9. doi: 10.1002/(sici)1520-6408(1996)18:2<>3.0.co;2-3 8934879

[14] Funakoshi Y Nakayama H Uetsuka K Nishimura R Sasaki N Doi K. Cellular proliferative and telomerase activity in canine mammary gland tumors. Vet Pathol. (2000) 37:177–83. doi: 10.1354/vp.37-2-177 10714647

[15] Lukens JN Van Deerlin V Clark CM Xie SX Johnson FB. Comparisons of telomere lengths in peripheral blood and cerebellum in Alzheimer's disease. Alzheimers Dement. (2009) 5:463–9. doi: 10.1016/j.jalz.2009.05.666, 19896585 PMC2859316

[16] Prasher B Negi S Aggarwal S Mandal AK Sethi TP Mukerji M . Whole genome expression and biochemical correlates of extreme constitutional types defined in Ayurveda. J Transl Med. (2008) 6:48. doi: 10.1186/1479-5876-6-48, 18782426 PMC2562368

[17] Juyal RC Negi S Wadhwa R Natu SM Singh S Mukerji M. Potential of ayurgenomics approach in predicting susceptibility to complex disorders. OMICS. (2012) 16:521–30. doi: 10.1371/journal.pone.0045752

[18] Govindaraj P Nair S Bhat BK Rotti H Bhale S Nayak J . Genome-wide analysis correlates Ayurvedic Prakriti. Sci Rep. (2015) 5:15786. doi: 10.1038/srep15786 26511157 PMC4625161

[19] Tiwari R Tiwari A Sharma V Singh A. Association between Prakriti and risk of type 2 diabetes: a cross-sectional observational study. J Ayurveda Integr Med. (2017) 8:266–75. doi: 10.1016/j.jaim.2017.05.004, 28869082 PMC5747506

[20] Shilpa S Venkatesha Murthy CG. Ayurvedic Prakriti and their cardiovascular disease risk profiles. Ayu. (2011) 32:494–9. doi: 10.4103/0974-8520.96122, 22661843 PMC3361924

[21] Mahalle N Kulkarni M Pendse N Naik S. Association of ayurvedic constitution (Prakriti) with arterial stiffness, inflammatory markers, and metabolic traits. Ayu. (2012) 33:238–42. doi: 10.4103/0974-8520.105244, 23559796 PMC3611644

[22] Charaka. Charaka Samhita, Vimana Sthana (Sharma RK, Dash V, translators). 8:95–99. Varanasi: Chaukhambha Sanskrit Series; (2014).

[23] Sushruta. Sushruta Samhita, Sharira Sthana (Srikantha Murthy KR, translator) 4:63–69. Varanasi: Chaukhambha Orientalia; (2012).

[24] Vagbhata. Ashtanga Hridaya, Sharira Sthana, Chapter 3. (Srikantha Murthy KR, translator). Varanasi: Chaukhambha Krishnadas Academy (2012).

[25] Cuenca-Zaldívar JN Del Corral-Villar C García-Torres S Araujo-Zamora R Gragera-Peña P Martínez-Lozano P . Fourteen-year retrospective cohort study on the impact of climatic factors on chronic musculoskeletal pain: a Spanish primary care analysis. Int J Rheum Dis. (2025) 28:e70125. doi: 10.1111/1756-185X.70125, 40040581

[26] Gaál ZA Boha R Stam CJ Molnár M. Age-dependent features of EEG-reactivity—spectral, complexity, and network characteristics. Neurosci Lett. (2010) 479:79–84. doi: 10.1016/j.neulet.2010.05.037, 20560166

[27] Biasiucci A Franceschiello B Murray MM. Electroencephalography. Curr Biol. (2019) 29:R80–5. doi: 10.1016/j.cub.2018.11.052, 30721678

[28] Niedermeyer E da Silva FL. Electroencephalography: Basic principles, clinical applications, and related fields. Philadelphia: Lippincott Williams & Wilkins (2005).

[29] Buzsáki G. Rhythms of the brain. Oxford: Oxford University Press (2006).

[30] Grandy TH Werkle-Bergner M Chicherio C Schmiedek F Lövdén M Lindenberger U. Peak individual alpha frequency qualifies as a stable neurophysiological trait marker in healthy younger and older adults. Psychophysiology. (2013) 50:570–82. doi: 10.1111/psyp.12043 23551082

[31] Klimesch W. An algorithm for the EEG frequency architecture of consciousness and brain body coupling. Front Hum Neurosci. (2013) 7:766. doi: 10.3389/fnhum.2013.00766 24273507 PMC3824085

[32] Alexander SPH Kelly E Marrion NV Peters JA Faccenda E Harding SD . The concise guide to pharmacology 2017/18: overview. Br J Pharmacol. (2017) 174:S1–S16. doi: 10.1111/bph.13882 29055037 PMC5650665

[33] Sharma R Dash B Dwivedi L. Ayurveda and geriatric health care: a comprehensive review. J Ayurveda Integr Med. (2015) 6:161–70.

[34] Niccoli T Partridge L. Ageing as a risk factor for disease. Curr Biol. (2012) 22:R741–52. doi: 10.1016/j.cub.2012.07.024 22975005

[35] World Health Organization. (2025). Ageing and health. Geneva, Switzerland: World Health Organization.

[36] OECD. (2025). Elderly population. Paris, France: Organisation for Economic Co-operation and Development (OECD).

[37] Murphy MC Mosler AB Rio EK Coventry M Raj IS Chivers PT . Can quantitative sensory testing predict treatment outcomes in hip and knee osteoarthritis? A systematic review and meta-analysis of individual participant data. Pain. (2025) 166:2261–80. doi: 10.1097/j.pain.0000000000003627, 40310871

[38] Russo A Borrelli F. *Bacopa monniera*, a reputed nootropic plant: an overview. Phytomedicine. (2005) 12:305–17. doi: 10.1016/j.phymed.2003.12.008 15898709

[39] Vijayan V Helen A. Protective activity of *Bacopa monniera* Linn. On nicotine-induced toxicity in mice. Phytother Res. (2007) 21:378–81. doi: 10.1002/ptr.2073 17236174

[40] Meléndez-Oliva E Martínez-Pozas O Sinatti P Martín Carreras-Presas C Cuenca-Zaldívar JN Turroni S . Relationship between the gut microbiome, tryptophan-derived metabolites, and osteoarthritis-related pain: a systematic review with meta-analysis. Nutrients. (2025) 17:264. doi: 10.3390/nu17020264, 39861394 PMC11767305

[41] Ornish D Lin J Chan JM Epel E Kemp C Weidner G . Effect of comprehensive lifestyle changes on telomerase activity and telomere length in men with biopsy-proven low-risk prostate cancer: 5-year follow-up of a descriptive pilot study. Lancet Oncol. (2013) 14:1112–20. doi: 10.1016/S1470-2045(13)70366-8, 24051140

[42] Karunaratne S Hanwella R De Silva V. Validation of the Sinhala version of the Montreal cognitive assessment in screening for dementia. Ceylon Med J. (2011) 56:147–53. 22298207 10.4038/cmj.v56i4.3892

[43] Hobson J. The Montreal cognitive assessment (MoCA). Occup Med. (2015) 65:764–5. 10.1093/occmed/kqv078 26644445

[44] Nasreddine ZS Phillips NA Bédirian V Charbonneau S Whitehead V Collin I . The Montreal cognitive assessment, MoCA: a brief screening tool for mild cognitive impairment. J Am Geriatr Soc. (2005) 53:695–9. doi: 10.1111/j.1532-5415.2005.53221.x 15817019

[45] O’Driscoll C Shaikh M. Cross-cultural applicability of the Montreal cognitive assessment (MoCA): a systematic review. J Alzheimer's Dis. (2017) 58:789–801. doi: 10.3233/JAD-161042, 28482634

[46] Rossetti HC Lacritz LH Cullum CM Weiner MF. Normative data for the Montreal cognitive assessment (MoCA) in a population-based sample. Neurology. (2011) 77:1272–5. doi: 10.1212/WNL.0b013e318230208a, 21917776

[47] Department of Ayurveda. Ayurveda Aushadha Sangrahaya. Colombo: Department of Ayurveda (1976).

[48] López-Otín C Blasco MA Partridge L Serrano M Kroemer G. The hallmarks of aging. Cell. (2013) 153:1194–217. doi: 10.1016/j.cell.2013.05.039, 23746838 PMC3836174

[49] Chung HY Kim DH Lee EK Chung KW Chung S Lee B . Redefining chronic inflammation in aging and age-related diseases: proposal of the Seno-inflammation concept. Aging Dis. (2019) 10:367–82. doi: 10.14336/AD.2018.0324, 31011483 PMC6457053

[50] Sanghavi T Govindaraj P Prasher B. Ayurgenomics: a systems biology approach for integrating Ayurveda and genomics. Front Genet. (2021) 12:644123. doi: 10.3389/fgene.2021.644123

[51] Fernández-Carnero S Martínez-Pozas O Cuenca-Zaldívar JN Sánchez-Romero EA. Addressing frailty in older adults: an integrated challenge for health, science, and society. Aging (Albany NY). (2024) 16:13176. doi: 10.18632/aging.206162, 39611815 PMC11719113

[52] Janković SM Bogavac‑Stanojević N Mikulić I Izetbegović S Iličković I Krajnović D . A questionnaire for rating health‑related quality of life. Slovenian Journal of Public Health. (2021) 60:260–8. Available online at: https://www.mendeley.com/catalogue/85d81d8c-2a2a-389f-927f-8d83f31cfd2c/?utm, 34917195 10.2478/sjph-2021-0035 PMC8643113

